# Metabolic Approaches for the Treatment of Dilated Cardiomyopathy

**DOI:** 10.3390/jcdd10070287

**Published:** 2023-07-05

**Authors:** Roberto Spoladore, Giuseppe Pinto, Francesca Daus, Sara Pezzini, Damianos Kolios, Gabriele Fragasso

**Affiliations:** 1 Department of Cardiology, Heart Failure Clinic, Alessandro Manzoni Hospital, ASST Lecco, 23900 Lecco, Italy; 2 IRCCS Humanitas Research Hospital, Rozzano, 20089 Milan, Italy; 3 Post-Graduate School of Cardiovascular Medicine, Milan-Bicocca University, 20126 Milan, Italy; 4 Department of Clinical Cardiology, Heart Failure Clinic, IRCCS San Raffaele Scientific Institute, 20132 Milan, Italygabriele.fragasso@hsr.it (G.F.)

**Keywords:** dilated cardiomyopathy, heart failure, metabolic therapy, myocardial energetics, SGLT2 inhibitors

## Abstract

In dilated cardiomyopathy (DCM), where the heart muscle becomes stretched and thin, heart failure (HF) occurs, and the cardiomyocytes suffer from an energetic inefficiency caused by an abnormal cardiac metabolism. Although underappreciated as a potential therapeutic target, the optimal metabolic milieu of a failing heart is still largely unknown and subject to debate. Because glucose naturally has a lower P/O ratio (the ATP yield per oxygen atom), the previous studies using this strategy to increase glucose oxidation have produced some intriguing findings. In reality, the vast majority of small-scale pilot trials using trimetazidine, ranolazine, perhexiline, and etomoxir have demonstrated enhanced left ventricular (LV) function and, in some circumstances, myocardial energetics in chronic ischemic and non-ischemic HF with a reduced ejection fraction (EF). However, for unidentified reasons, none of these drugs has ever been tested in a clinical trial of sufficient size. Other pilot studies came to the conclusion that because the heart in severe dilated cardiomyopathy appears to be metabolically flexible and not limited by oxygen, the current rationale for increasing glucose oxidation as a therapeutic target is contradicted and increasing fatty acid oxidation is supported. As a result, treating metabolic dysfunction in HF may benefit from raising ketone body levels. Interestingly, treatment with sodium-glucose cotransporter-2 inhibitors (SGLT2i) improves cardiac function and outcomes in HF patients with or without type 2 diabetes mellitus (T2DM) through a variety of pleiotropic effects, such as elevated ketone body levels. The improvement in overall cardiac function seen in patients receiving SGLT2i could be explained by this increase, which appears to be a reflection of an adaptive process that optimizes cardiac energy metabolism. This review aims to identify the best metabolic therapeutic approach for DCM patients, to examine the drugs that directly affect cardiac metabolism, and to outline all the potential ancillary metabolic effects of the guideline-directed medical therapy. In addition, a special focus is placed on SGLT2i, which were first studied and prescribed to diabetic patients before being successfully incorporated into the pharmacological arsenal for HF patients.

## 1. The Purpose of Metabolic Therapy in Dilated Cardiomyopathy

Dilated cardiomyopathy (DCM) is a primary myocardial disorder characterized by enlarged ventricles with a contractile deficit that leads to reduced ventricular function in the absence of volume or to pressure overload, congenital heart disease (CHD), or significant coronary artery disease (CAD) [1]. Genetic and non-genetic causes underlie its pathogenesis, in which a mixed mechanism is often involved [2]. Its estimated prevalence in the general population ranges from 1:500 to 1:2500 [3,4].

DCM is a recognized cause of systolic heart failure (HF), a condition in which the impaired cardiac pump is unable to fulfil the energetic demand of the body in terms of nutrients and oxygen supply. HF has been defined as the cardiovascular epidemic of the 21st century [5], with an estimated prevalence of about 2–4% in the general adult population; this prevalence is known to increase with age [6,7]. Moreover, due to the prevalence of HF in high-income countries, significant amounts of human and economic resources are employed in this field [8].

Multiple factors, such as hemodynamics, neurohormones, and genetics, participate in progressive heart failure remodeling [9]. The efficiency of the myocardial pump depends mainly on the metabolism of the cardiac cells [10]. However, the hyperactivation of the adrenergic system and the renin–angiotensin–aldosterone system (RAAS), when attempting to sustain hemodynamic failure, contributes to indirect changes in the metabolism of the cardiac and skeletal muscles, worsening their efficiency [11]. In the last decades, better knowledge of the pathophysiological mechanisms of the effects of the neurohormonal axis on the cardiovascular (CV) system has enabled the adoption of drugs that block this detrimental activation. Despite the development of additional molecules targeting different pathological pathways, as outlined by the European and American guidelines on HF [12,13], a specific phenotype-oriented therapy is generally lacking, and the HF prognosis remains poor [14].

In the last decades, there has been a growing interest in the cardiac metabolism; the aim has been to find new potential disease biomarkers and therapeutic targets to improve the HF prognosis [15].

A healthy adult heart produces adenosine triphosphate (ATP) by metabolizing different types of fuels (fatty acids, glucose, lactate, ketones, and amino acids), primarily via oxygen-dependent mitochondrial oxidative phosphorylation and the electron transport chain and, to a lesser extent, the anaerobic glycolytic pathway [16]. Between 60 and 80% of the energy produced by the healthy heart is derived from free fatty acid (FFA) oxidation, despite a metabolic flexibility that allows a shift between different energy substrates to maintain ATP production [16]. However, FFA oxidation is a less efficient source of energy production than glucose oxidation (in terms of the produced ATP per consumed O2 molecules). Of note, the amount of ATP produced per O2 molecules consumed is greater for glucose oxidation compared to that of FFAs. For example, the mitochondrial ATP yield per oxygen atom (P/O ratio) is only 2.33 for long-chain fatty acids, whereas it is 2.50 for ketone bodies and 2.58 for glucose. Consequently, oxidizing glucose results in an increase in cardiac efficiency of up to 30% [17].

The failing heart is considered to be an “engine out of fuel” [18]. A reduction in the mitochondrial oxidative capacity is the first metabolic change characterizing the heart’s deteriorating energy deficiency [19,20]. The ensuing increased glycolytic pathway [21] is not as efficient as mitochondrial phosphorylation and fails to compensate for the status of the energy deficit; this also leads to H^+^ accumulation in the cytoplasm as glycolysis is uncoupled from the oxidation of pyruvate and lactate [22,23]. Moreover, a highly significant shift in substrate utilization also occurs. A decrease in FFA oxidation has been observed in humans with idiopathic DCM [24], despite evidence from other studies that is not consistent with this finding [25,26]. Furthermore, despite a reduction in FFA oxidation, the failing heart still mainly counts on this substrate for the highest proportion of mitochondrial ATP generation [27].

Changes in substrate utilization in the failing heart have also been observed relative to glucose metabolism, ketone bodies, and long-chain amino acids [15]. More metabolites have been found to be involved in the pathophysiological process of HF; thus, the studies on HF are no longer limited to glucose and FA metabolism [28]. When glucose and lipid metabolism decrease, it is possible to hypothesize that ketone bodies may act as alternative substrates in failing hearts [28]. Due to insulin resistance and other factors, the failing heart has significantly reduced glucose and fatty acid utilization, and ketone bodies are fast-metabolizing small molecular energy substrates that the heart can use to improve cardiac efficiency. In addition, recent research has demonstrated that branched-chain amino acids (BCAAs) also play a significant role in the pathophysiology of end-stage heart failure. The myocardial BCAA metabolism can effectively improve cardiac function and slow the progression of heart failure [28]. Therefore, the metabolic remodeling of small molecular substrates such as ketone bodies and amino acids also plays a significant role in the onset and progression of HF, in addition to the changes in the metabolism of glucose and FAs during HF. However, there have been conflicting results; these results are partially explained by the disparities in the severity of the disease and the presence of other medical conditions, such as metabolic syndrome, in the individuals participating in human clinical studies.

In addition, in systolic heart failure, metabolic changes can occur regardless of weight status. However, the specific metabolic changes seen in obese, normal-weight, and underweight individuals may vary. There may be an increased reliance on fatty acid oxidation as a source of energy for the heart in obese people with heart failure. This can lead to an impaired glucose metabolism and decreased myocardial glucose uptake and oxidation. In addition, metabolic dysregulation in the heart may be exacerbated by insulin resistance and inflammation, which are frequently associated with obesity. As previously mentioned, heart failure can cause metabolic changes, such as an increase in glycolysis as a source of myocardial energy, even in healthy people. In heart failure, the oxidative metabolism is less efficient; so, this metabolic switch is made to make up for it. Heart failure patients who are underweight may experience metabolic changes that are comparable to those seen in people of normal weight. There may still be a shift toward more glycolysis [29]. Implementing strategies for the targeted reduction in this particular fat store in obese individuals is the obvious solution to the problem of preventing adipose tissue inflammation and the metabolic and cardiovascular complications that come with it. The treatment for insulin resistance and obesity still relies heavily on lifestyle changes, such as diet and exercise modifications [29].

Recently, the use of various “omics” technologies, such as metabolomics, has offered a new chance to enhance our understanding of the mechanisms involved in this disease and to find new biomarkers for the prognosis and diagnosis of DCM [30]. A recent comprehensive review on this topic highlighted what the recent literature has provided in terms of the metabolite-based biomarkers which are useful for predicting and diagnosing DSM and for monitoring therapeutic interventions [31]. One main limitation in understanding metabolic pathophysiology from the results of these studies is their limited sample sizes, which prevent the reaching of a definitive conclusion about the practicality of the identified DCM biomarkers for clinical purposes. Moreover, since these studies have mainly been conducted on biofluids such as serum, the alterations in plasma metabolites may represent the impact of the contribution of several organs. To overcome this last issue, a recent analysis by Flam et al. was conducted on myocardial biopsies from patients with end-stage HF; the analysis comprised metabolomics, genome-wide RNA sequencing, and global proteomic assessment [32]. The findings confirmed the significant alterations in the metabolic process of the heart that had been seen previously in HF experiments with animal subjects, including a decline in the utilization of fatty acids and a heightened dependence on the utilization of ketones and carbohydrates. These results aligned with prior studies on human and animal models of HF. However, this particular study provides a novel insight. The previous research on animal models suggested that the decrease in fatty acid oxidation was due to a decline in mitochondrial oxidative function. However, the present study revealed a scarcity of fatty acids or acylcarnitines in the damaged heart, rather than an accumulation, indicating a shortage of fatty acid supply to the heart. As plasma fatty acid levels in HF patients remain unchanged, this scarcity in heart tissue could suggest a potential issue with fatty acid import. The specific cause of this is still unknown. This discovery is significant because it provides a new avenue for the targeting of the fatty acid metabolism in the treatment of HF. It is essential to determine whether similar changes occur during the development of HF and not just as a result of advanced disease. The study found that multiple classes of carbon substrates, including many amino acids, tricarboxylic acid cycle (TCA) metabolites, and glycolytic intermediates, were all reduced in the failing heart samples. This raises the possibility that these changes may reflect a state of malnutrition in end-stage disease and must be ruled out. Another difference between the human and animal studies is that HF patients often receive extensive treatment, which may impact the cardiac metabolism. For example, betablocker treatment could significantly reduce fatty acid uptake in failing hearts [33].

Furthermore, Zhao et al., in a study conducted on patients’ plasma, showed that both DCM and ischemic cardiomyopathy (ICM) patients have a unique metabolomics profile compared to that of healthy controls, with some common metabolic changes [34]. Additionally, the study revealed that DCM and ICM patients display different metabolomics patterns, with specific metabolic pathways implicated in each disease, emphasizing the importance of metabolic imbalances in distinct disease mechanisms [34].

**Figure 1:**
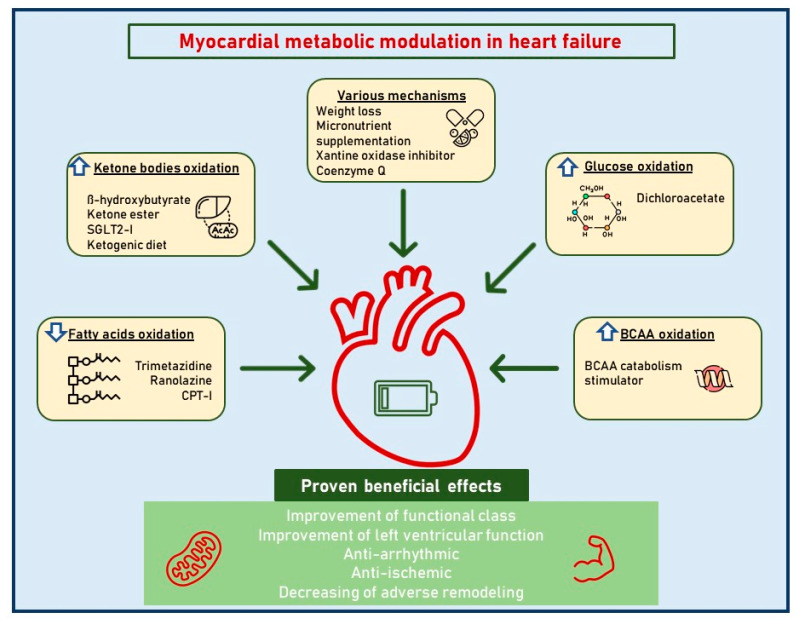
Myocardial metabolic modulation in dilated cardiomyopathy. This figure illustrates the different therapeutic metabolic targets in dilated cardiomyopathy. Abbreviations: SGLT2i, sodium-glucose cotransporter-2 inhibitors; BCAAs, branched-chain amino acids.

A better knowledge of cardiac metabolic adaptations in HF would certainly serve as a starting point from which to highlight new therapeutic targets, for old and new drugs, to exploit in different phenotypes and disease stages (Figure 1; Table 1).

## 2. Metabolic Effects of Neurohormonal Hyperactivation Antagonists

### 2.1. Betablockers

Without affecting their detrimental chronotropic and inotropic effects, betablockers have the ability to directly alter myocardial energetics [35]. By reducing peripheral lipolysis, this pharmacological class lowers the levels of FFA in the blood and allows a change in the heart’s energy metabolism that increases the use of carbohydrates [36]. Through these metabolic processes, betablockers are responsible for this substrate competition’s decreased myocardial FFA uptake and increased glucose utilization [37,38]. A decrease in FFA delivery and an increase in the availability of arterial glutamate, which is highly advantageous for myocardial tissue as it can serve as both aerobic and anaerobic fuel, making it a particularly versatile substrate [38], are both likely to be responsible for the increase in carbohydrate metabolism in the heart that was observed after a beta-blockade [39,40]. As a result, a beta-blockade may result in higher glucose consumption, which in turn may cause the heart to produce more energy without using up more oxygen. This suggests that in addition to their hemodynamic effects, betablockers may also directly affect the metabolic alterations observed in heart failure. When betablockers are used to treat systolic HF, greater energy efficiency and decreased oxygen consumption are seen. The changes in the way the heart produces energy may be the cause of these changes [41]. In actuality, the phenomenon of heart-rate reduction in HF patients may only be a marker of a greater functional response to betablocker therapy [42]. To lessen the failing heart’s reliance on fatty acids and to overcome the inhibition of myocardial glucose utilization brought on by fatty acids, the primary goal of therapy may be to reduce the plasma levels of FFAs and triacylglycerols. According to two studies, patients with New York Heart Association (NYHA) functional class III HF who took the betablocker carvedilol had lower FFA utilization and higher glucose utilization [43,44]. The reduction in oxygen consumption and the increase in energy effectiveness seen in HF patients after betablocker medication could possibly be explained by a change in the way the heart produces energy. In shifting the body’s energy substrate usage from lipid to glucose oxidation, non-selective betablockers appear to be more successful than selective ones [44]. Non-selective betablockers, on the other hand, appear to worsen insulin resistance, which is already known to be linked to HF and CV disorders [45,46]. However, it does not seem that vasodilators and cardio-selective drugs promote insulin resistance. Carvedilol, in particular, may have beneficial metabolic effects on boosting insulin sensitivity in HF patients [47]. Notably, the former’s greater metabolic effects may be one of the causes of the higher survival rates seen during their usage [48].

### 2.2. RAAS Inhibitors

The hormone system known as RAAS controls blood pressure and fluid balance. Angiotensin I (AT I) conversion to angiotensin II (AT II) is blocked by RAAS inhibitors, and the AT II receptors at the end of the route are also blocked. Due to its direct ability to cause and sustain ventricular dysfunction through a variety of pathways, AT II is a key participant in the regulation of cardiac energy metabolism [49]. It affects mitochondrial oxidative phosphorylation, particularly fatty acid oxidation [50], and damages cardiomyocyte mitochondria by increasing the formation of reactive oxygen species [51]. Additionally, there is proof that AT II reduces glucose oxidation [52]. Overall, AT II can lower ATP levels by reducing oxidative metabolism [53]. Its antagonism represents an appealing therapeutic strategy in this situation. Studies using the euglycemic insulin clamp technique demonstrated that the positive impact of AT II antagonistic action is exerted on insulin sensitivity. In fact, it has been demonstrated that ACE inhibitors [54] and angiotensin receptor antagonists [55] improve glucose homeostasis and left ventricular performance. The potential routes of action include elevated skeletal muscle blood flow, bradykinin build up, or more effective insulin release. Finally, RAAS inhibitors are able to reduce the atrial wall stress and fibrosis, consequently promoting a progressive reverse remodeling of the enlarged left atrium in dilated cardiomyopathy, with positive outcome effects [56].

### 2.3. Angiotensin Receptor Neprilysin Inhibitors (ARNI)

After a median follow-up of 27 months, the PARADIGM-HF study demonstrated that sacubitril/valsartan, the first member of a new class of medications known as ARNI, reduced the morbidity and mortality of patients with HF and reduced EF compared with the ACE inhibitor enalapril [57]. Sacubitril/valsartan is thought to provide an extra benefit over the renin–angiotensin blockade alone because it inhibits neprilysin, an endopeptidase that breaks down endogenous vasoactive peptides such natriuretic peptides [58]. Although there were very few patients who developed new-onset diabetes during the course of the PARADIGM-HF trial, sacubitril/valsartan did not lower the pre-specified exploratory outcome of new-onset diabetes, in contrast to enalapril. Despite this, new research suggests that sacubitril/valsartan, regardless of diabetes, may enhance lipid metabolism, insulin sensitivity, and glucose metabolism in individuals with HF [59,60].

### 2.4. Mineralocorticoid Receptor Antagonists (MRAs)

Aldosterone has negative effects that are mediated via the mineralocorticoid receptor; these effects are blocked by MRAs such as spironolactone and eplerenone. MRAs are therefore effective in treating hypertension, especially resistant hypertension, and in lowering the risk of morbidity and death in HF patients through this pharmacological activity. The “off-target effects” of spironolactone have also been shown to have negative effects on lipid and glucose homeostasis [61]. The blockage of glucocorticoid receptors by spironolactone is thought to be the mechanism by which cortisol blood concentrations are raised. By accelerating lipolysis and gluconeogenesis, the glucocorticoid cortisol raises blood glucose levels. On the other hand, the selective MRA eplerenone has relatively little effect on other steroid receptors. Because of this, it does not impact glucose metabolism, and it lowers serum cortisol levels [61]. It has been confirmed that spironolactone may cause changes in blood glucose levels, whereas eplerenone has no effect on glucose homeostasis, according to a recent systematic review of randomized controlled trials, prospectives, and observational studies evaluating the influence of the various MRAs on the biomarkers of glucose homeostasis in a variety of populations [62,63]. The Eplerenone in Mild Patients Hospitalization and Survival Study in Heart Failure (EMPHASIS-HF) study of participants with chronic HF actually had no effect on new-onset diabetes [64].

### 2.5. Loop Diuretics

Loop diuretics, such as furosemide, primarily affect the kidneys to increase urine production. However, they may also have an indirect effect on the heart’s metabolism. Hypomagnesemia and hypokalemia can be brought on by loop diuretics. Arrhythmias are more likely to occur in people with these two conditions, which can impair cardiac function by interfering with normal electrical signaling. Additionally, hypocalcemia, which may affect cardiac contractility, can be brought on by these medications. Last but not least, loop diuretics can contribute to all of the metabolic changes associated with RAAS activation (as previously described) by stimulating the RAAS through volume depletion and decreased blood pressure [65].

## 3. Direct Cardiac Metabolism Modulators

Although the ideal metabolic environment for a failing heart remains highly debated and poorly understood, it may be a good target for future treatments. With the use of medications such as trimetazidine, ranolazine, perhexiline, and etomoxir, the previous research has concentrated on boosting glucose oxidation, which, in comparison to FFA oxidation, has a higher P/O ratio. These medications have been shown to enhance myocardial energetics and LV function in patients with chronic ischemic and non-ischemic HF and low EF. For unknown reasons, these medications have not, however, ever undergone extensive clinical studies [66].

The failing heart in severe non-ischemic cardiomyopathy, on the other hand, may be metabolically adaptive and not oxygen-restricted, according to some preliminary investigations. This challenges the conventional wisdom that the optimal therapeutic goal is to increase glucose oxidation; instead, it supports the alternative position that increasing fatty acid oxidation is a wise course of action [66].

### 3.1. Trimetazidine

Trimetazidine is an anti-ischemic medication that, as a result of the inhibition of mitochondrial long-chain 3-ketoacyl CoA thiolase, directly reduces cardiac fatty acid oxidation. This leads to a better coupling of glucose oxidation with glycolysis, which could account for its antianginal effect [67].

It has been demonstrated that this drug also maintains the intracellular levels of high-energy phosphate in the myocardium, improving the ratio of phosphocreatine to ATP by a net 33%. Previous research has revealed that this ratio, which is utilized as a measure of cardiac energetics, is decreased in the failing human myocardium [68]. In a trial on HF patients on trimetazidine, the improvement of this ratio, which has been shown to be a major predictor of mortality [69], led to various positive effects, including improvements in functional class, exercise performance, and LV function [70].

Additionally, it has been shown that trimetazidine has a positive impact on the vascular endothelium, as well as glucose metabolism in T2DM patients with ischemic cardiomyopathy, by decreasing endothelin-1 release and increasing insulin-induced glucose oxidation and cyclic guanosine monophosphate (cGMP) release from skeletal muscle cells [71]. Trimetazidine appears to be a viable therapeutic option because poor glucose metabolism in individuals with HF contributes unquestionably to decreased cardiac efficiency.

In fact, a multicenter retrospective cohort analysis on 669 HF patients revealed that the addition of trimetazidine to standard medical care improved HF hospitalizations and survival [72]. In addition, four meta-analyses examined the possibility that trimetazidine, in addition to the best possible medical care, improved the chances of survival in HF patients [73,74,75,76]. Overall, this medication improved event-free survival and decreased all-cause death in HF patients. However, the sample size for the clinical trials and the observational studies conducted on this medication were extremely limited, and as a result, a small number of patients were included in the meta-analysis, making it impossible to draw firm conclusions.

Finally, a recent study has shown that a new molecule called ninerefaxstat considerably improves myocardial energetics, lowers myocardial steatosis, and improves diastolic function in individuals with T2DM and obesity [77]. This substance releases a trimetazidine analogue after fast hydrolysis. Future research is required to show whether this unique metabolic substrate changer has any positive impact on patients with DCM and HF.

### 3.2. Ranolazine

A piperazine-derived substance called ranolazine selectively inhibits the late sodium current in cardiomyocytes. Ischemia, HF, and other pathological circumstances can increase channel activity, which, in turn, causes a reduction in systolic force generation and an increase in diastolic force generation, as well as an increase in oxygen consumption [78].

By lowering sodium influx, ranolazine prevents calcium overload and the ensuing rise in wall tension. This would seem to be its primary antianginal mechanism [79]. The beneficial effects of ranolazine may possibly result from the promotion of glucose oxidation and the inhibition of fatty acid oxidation, which improves the ratio of phosphocreatine to ATP and lowers the levels of H+, lactate, and toxic fatty acyl intermediates [80,81]. However, particularly in HF patients, its potential as a metabolic modulator has not yet been explored.

### 3.3. Perhexiline and Etomoxir

Carnitine-palmitoyltransferase (CPT-I), an enzyme involved in the absorption of mitochondrial fatty acids, is inhibited by perhexiline. Its inhibition increases glucose oxidation while decreasing fatty acid oxidation [66]. In patients with chronic HF, perhexiline has been demonstrated to increase exercise capacity, LV EF, and symptoms [80]. In a randomized experiment with non-ischemic HF patients, perhexiline has recently been shown to enhance cardiac energy status by improving the phosphocreatine/ATP ratio by up to 30%, with no evidence of altered cardiac substrate usage. Additionally, it demonstrated a notable improvement in functional status [82,83].

Etomoxir inhibits CPT-I, which has comparable effects on glucose metabolism to perhexiline. Additionally, etomoxir may directly activate PPAR (peroxisome proliferator activated receptor) alpha, which would upregulate a number of beta-oxidation-related enzymes [84]. In two distinct experiments using diabetic rats, this medication markedly enhanced cardiac function [85].

Furthermore, etomoxir added to standard therapy improved LV EF, central hemodynamics at rest and during exercise, and clinical status in a small pilot study with ten patients with HF (one patient had ischemic heart disease and nine had dilated idiopathic cardiomyopathy) [86].

Despite these findings, etomoxir has a limited therapeutic application since it may lead to heart hypertrophy [87] and because it has been reported to generate oxidative stress and disrupt mitochondrial energy metabolism [88,89].

Additionally, the elevated liver transaminase levels of four patients caused the ERGO study, which was intended to verify the effectiveness of 80 mg of etomoxir/day, to be discontinued early [90].

### 3.4. Other Potential Therapeutic Strategies

A key cofactor of fatty acid metabolism, L-carnitine, which is a modified amino acid that resembles a vitamin, controls the intramitochondrial acyl-coenzyme A/coenzyme A ratio. Although its primary function is to support FFA metabolism, there is some evidence from in vitro studies that it can also improve glucose metabolism. A minor improvement in heart energetics and function with L-carnitine supplementation has been supported by a number of research studies in both human and animal tests [91]. Despite this, its therapeutic usefulness as a nutritional supplement for ischemic heart disease and heart failure was never established. L-carnitine supplementation is helpful in HF patients in reducing symptoms and cardiac functioning and in decreasing blood biomarkers, but it does not give a revival advantage to these patients, according to a recent meta-analysis involving 1625 individuals with chronic systolic HF [92].

The reduction in reactive oxygen species caused by xanthine oxidase inhibition [93] may also have an impact on heart energetics. In both HF animal models and human patients with persistent systolic HF, it was demonstrated that the xanthine oxidase inhibitor allopurinol enhanced cardiac efficiency [94,95]. Allopurinol, however, did not enhance clinical status, exercise capacity, quality of life, or left ventricular ejection fraction when tested on hyperuricemic chronic systolic HF patients in a clinical trial [96].

It has been demonstrated that coenzyme Q, commonly known as ubiquinone, improves mitochondrial respiration [97], endothelial dysfunction, and cardiac contractility in patients with chronic HF [98]. In a clinical trial, coenzyme Q treatment in HF patients with impaired EF was found to be safe, to improve symptoms, and to lower significant adverse cardiovascular events [99]. A recent state-of-the-art review examined its potential role as a future therapy for HF, outlining the limitations resulting from earlier studies, such as the small sample size of the population studied; requesting future studies with adequate power to assess the clinical benefit of ubiquinone in patients with HF and reduced EF; and speculating on a potential role for HF patients with preserved EF [100].

Overcoming the failing heart’s insulin resistance state is another possible strategy for increasing metabolic efficiency. In various circumstances, the direct pyruvate dehydrogenase kinase inhibitor dichloroacetate (DCA) has been shown to improve glucose oxidation. Pre-clinical research on HF animal models revealed that DCA therapy could increase contractile reserve and stop the HF rat from decompensating [101,102]. Additionally, DCA was demonstrated to increase myocardial mechanical efficiency and glucose oxidation in patients with congestive HF (NYHA classes III–IV) by lowering myocardial oxygen consumption [103].

Branched-chain amino acids (BCAAs) provide an additional energy source for the failing heart. Both the plasma and the heart contained higher concentrations of BCAAs and their metabolites in HF rat models [104,105]. In addition, both the administration of BCAAs and the suppression of BCAA catabolism had a negative impact on cardiac function in preclinical trials [104,106]. Interestingly, the left ventricular samples from patients with dilated cardiomyopathy undergoing heart transplantation had higher cardiac BCAA levels than did those from the healthy controls, and a BCAA catabolism stimulator significantly improved cardiac function in HF mouse models [105]. However, the precise mechanism by which impaired BCAA oxidation contributes to the onset and/or severity of decreased oxidative glucose metabolism is still unknown. Oxidative phosphorylation in mitochondria, which is tightly controlled by the turnover of the ATP that powers cardiac contraction and relaxation, meets the heart’s enormous energy needs. This mechano-energetic coupling is disrupted in heart failure (HF), resulting in a bioenergetic mismatch and the production of ROS that accelerate cardiac dysfunction [107]. Although treatment with unspecific and untargeted antioxidants failed to produce the anticipated results, targeting ROS in cardiovascular diseases has long been regarded as a promising therapeutic strategy [108]. Elamipretide is a small, cell-permeable peptide that targets cardiolipin in particular [107]. Cardiolipin is a phospholipid that only lives in mitochondria, where it is crucial to the structural and functional organization of the macromolecular complexes embedded in the inner mitochondrial membrane. For instance, cardiolipin stabilizes the supercomplexes of the respiratory chain complexes for optimal oxidative phosphorylation. In animal models of myocardial infarction and ischemia–reperfusion injury, preclinical studies with elamipretide demonstrated improved left ventricular (LV) contractile function and a smaller infarct size. Elamipretide was safe and well tolerated in humans. Elamipretide is still being evaluated in clinical trials, however [107].

Last, but not least, lactate is a normal fuel for the heart and can be converted into pyruvate by the enzyme lactate dehydrogenase (LDH) and fed into the tricarboxylic acid (TCA) cycle, which in turn produces ATP [108]. Although it is commonly thought of as a waste product, it was recently reported that lactate, an important energy source for the myocardium, feeds the TCA cycle through glucose [108]. In healthy human hearts, the simultaneous production of lactate from glycolytic pyruvate compensates for this lactate consumption [109]. However, when glycolytic pyruvate is preferentially converted to lactate during HF, this equilibrium is disrupted, resulting in a decrease in lactate consumption at the same time. The mitochondrial pyruvate carrier (MPC) is a heterodimeric complex of MPC1 and MPC2 that transports the byproduct of glycolysis, pyruvate, into the mitochondria, where it can be fully oxidized by pyruvate dehydrogenase (PDH) and the TCA cycle. The normal adult heart expresses the MPC subunits extensively [108]. Cardiomyocytes, on the other hand, express low levels of MCT4, which belongs to the monocarboxylate transporter (MCT) family and is thought to be the primary cell lactate exporter [109]. The MPC is likely a crucial control point at which the choice between pyruvate oxidation and cytosolic lactate conversion is made, as demonstrated in other cell types. Changing the pyruvate–lactate axis is a fundamental and early feature of cardiac hypertrophy and failure, as evidenced by the fact that highly potent MCT4 inhibition can reduce cardiac hypertrophy [110]. Future devices that interact with this axis may be useful in heart failure treatment.

## 4. The Ketone Bodies Hypothesis

As outlined above, targeting cardiac metabolism by decreasing fatty acid oxidation and promoting glucose oxidation appears to be an interesting approach to the treatment of chronic HF. Several drugs have been investigated in small-scale studies, but large clinical trials are needed to confirm the efficacy of these agents as a part of chronic HF treatment.

More recently, a new interest in ketone body metabolism has arisen as their modulation may be of potential benefit to HF patients.

Under normal conditions, ketones represent a minimal part of all substrates utilized by the myocardium for energy production. These compounds, however, become critical during periods of stress and fasting since their utilization allows the preservation of glycogen stores. The myocardium is the highest ketone body consumer per unit mass. Ketone body oxidation is also more efficient than fatty acid oxidation in terms of ATP synthesis per molecule of oxygen used [111,112]. In addition, ketone body metabolism exerts anti-oxidant effects since it oxidizes mitochondrial co-enzyme Q and reduces cytosolic [NADP+]/[NADPH+], thereby decreasing free radical production [112]. The resourcefulness of the heart in using ketone bodies in order to satisfy its ATP requirements serves as a tool to spare glucose. However, it remains rather unclear whether their employment is compensative to balance out the negative effects of the failing heart adaptive/maladaptive substrate utilization.

In hypertrophied and early-stage failing rat hearts, a reduced capacity to oxidize fatty acids and a shift to ketone oxidation as an alternative metabolic fuel have been observed [113]. Similar data have been found in failing human hearts: patients with reduced LV EF nearly tripled their consumptions of ketones as metabolic substrates compared to patients with preserved EF [27].

A case control study involving patients with chronic dilated non-ischemic cardiomyopathy showed increased amounts of beta-hydroxybutyryl CoA and decreased amounts of myocardial beta-hydroxybutyrate in myocardial tissue, suggesting an increased ketone body metabolism in this setting [114].

Additional studies have shown that circulating ketone bodies in subjects with chronic HF increase proportionally to the intensity of their symptoms, the level of congestion in the venous circulatory system, and the magnitude of neurohormonal and cytokine involvement, as well as the increasing deterioration of left ventricular function [115,116]. In this context, more ketones are produced through hepatic ketogenesis and become a fundamental substrate for energy production in cardiomyocytes [117].

These results clearly indicate that chronic HF determines a ketosis-prone state [115]. Indeed, exhaled acetone levels have been shown to be able to identify HF patients with a predictive value which is somewhat similar to that of brain natriuretic peptide (BNP); moreover, this predictive value is proportional to the NYHA class [116].

It is also known that exhaled breath acetone is increased in HF patients with reduced EF and is associated with higher mortality or heart transplantation [118].

Interestingly, higher serum levels of beta-hydroxybutyrate seem to relate to disease progression and adverse prognosis in arrhythmogenic cardiomyopathy patients, supporting the hypothesis that an enhanced ketone body metabolism may be a standard myocardium response to injuries [119].

According to another study, the cardiomyocytes’ specific loss of succinyl-CoA:3-oxoacid CoA transferase, which is involved in ketone body oxidation, is associated with significantly increased left ventricular volume and a decreased ejection fraction as a response to pressure overload [120]. Overall, these studies confirm the fundamental role of this metabolic pathway, showing that impaired ketone body oxidation may be associated with worsened heart remodeling following pressure overload.

In this context, the concept of the therapeutic modulation of ketone metabolism as a potential new target in HF treatment is emerging [120,121,122,123,124].

## 5. Sodium-Glucose Cotransporter-2 Inhibitors (SGLT2i)

There is a bidirectional link between diabetes mellitus (DM) and HF. Longstanding diabetes causes changes in myocardial metabolism, abnormal calcium signaling, and inflammatory pathways, resulting in structural and functional changes in the myocardium and leading to the development of diabetic cardiomyopathy and the progression of HF [125,126]. Conversely, HF patients without DM are at an increased risk of developing glycemic abnormalities [125]. The shared underlying risk factors and the overlap of the pathophysiological mechanisms play a critical role in the frequent coexistence of DM and HF. As with HF, there is also a strong link between diabetes, coronary artery disease, hypertension, and renal disease.

During the last decade, cardiovascular outcome trials have investigated several classes of new glucose-lowering agents, including SGLT2i, which, apart from showing evidence of cardiovascular safety, have also been shown to exert beneficial effects on the cardiovascular outcome [127,128]. Most studies have shown the independence of cardiovascular outcome from glycemic control, indicating mechanisms of action other than those usually postulated to explain the cardiovascular benefits of glucose-lowering therapies [129,130,131,132,133,134]. In fact, the significant beneficial clinical effects observed with SGLT2i use cannot be explained by one single mechanism.

### 5.1. Glucose Metabolism

SGLT2i increase urinary glucose excretion by suppressing the activity of SGLT2 protein in the nephron proximal convoluted tubule, lowering the maximal renal transport capacity for glucose reabsorption and, consequently, the threshold for glycosuria [135]. The ensuing caloric depletion due to glycosuria stimulates weight loss [136]. Additionally, SGLT2i increase insulin sensitivity, a mechanism that stimulates ketogenesis and glucose uptake in the muscle cells and inhibits gluconeogenesis [137]. In addition to glycosuria, SGLT2i also promote natriuresis and uricosuria [136]. Finally, it was found that the failing myocardia of non-diabetic pigs benefited significantly from an increase in the utilization of BCAAs through SGLT2 inhibition [138]. Furthermore, Kappel et al. [139] used an untargeted metabolomics approach and found that in T2DM and CVD patients, SGLT2i increased the degradation products of BCAAs. However, the effect of SGLT2 inhibitors on BCAA metabolism requires additional experimental research to clarify the role of BCAAs in cardiac protection.

### 5.2. Renal Effects

Numerous studies suggest a nephroprotective effect of long-term therapy with SGLT2i. In the CREDENCE trial, 4401 T2DM patients with an estimated glomerular filtration rate (eGFR) from 30 to 90 mL/min/1.73 m^2^ and albuminuria were randomly given canagliflozin or a placebo. Canagliflozin substantially reduced the risk of end-stage kidney disease and renal or cardiovascular death [140]. Of note, dapagliflozin and empagliflozin, in DAPA-CKD and EMPA-KIDNEY trials, respectively, reduced the risk of kidney failure in chronic kidney disease (CKD) patients with or without T2DM, independently of a history of HF [141,142]. As CKD is prevalent and associated with high mortality in HF, prevention of the progression and/or worsening of CKD needs to be considered as an important goal that may translate into improved outcomes in HF.

The mechanisms underlying the nephron protection of SGLT2i are not fully understood. In diabetic patients, glomerular hyperfiltration is an important mechanism of nephropathy. SGLT2i, by activating the tubulo-glomerular feedback, cause vasoconstriction of the afferent arteriole, resulting in a reduction in intraglomerular pressure and, consequently, glomerular hyperfiltration [143]. Reductions in intraglomerular hypertension may explain the significant long-term renal preservation noted with SGLT2i [135]. Moreover, compared with classical diuretics, the fluid loss mediated by SGLT2i causes an important reduction in interstitial fluid without a significant reduction in intravascular volume [135]. The ability to selectively reduce interstitial fluid may be of particular importance in patients with HF in whom intravascular contraction is often present. Furthermore, this mechanism could limit the reflex neurohumoral stimulation that occurs in response to intravascular volume contraction [135].

Of note, ACE-I and angiotensin receptor blockers (ARBs) cause efferent arteriolar vasodilatation and, when used in combination with SGLT2i, will likely co-impact on intraglomerular pressure and may account for the initial drop in eGFR observed in patients, which is followed by a plateau over time [135].

### 5.3. Cardiovascular Effects

So far, the mechanisms underlying the protective cardiovascular effects of SGLT2i in patients with and without T2DM are not completely understood, and several hypotheses have been proposed. SGLT2i may modulate many of the important underlying cellular mechanisms that have been documented to contribute to cardiac pathologies, such as hypertrophy, HF, diastolic dysfunction, and arrhythmias [144]. Large clinical trials involving patients with T2DM have shown that SGLT2i reduce the risk of HF hospitalization in patients with and without the disease at the baseline [133,145,146]. DAPA-HF and EMPEROR-Reduced were placebo-controlled trials designed to study the effects of dapagliflozin and empagliflozin, respectively, in patients with established HF and a reduced EF, regardless of the presence of T2DM. The results confirmed that among patients with HF and a reduced EF, those who received SGLT2i had a lower risk of worsening HF or of death from cardiovascular causes [147,148]. Furthermore, the long-term use of SGLT2i (8 months in DAPA-HF trial) in patients with HF seems to improve their quality of life, as measured by the Kansas City Cardiomyopathy Questionnaire (KCCQ) [149].

Several mechanisms other than improved glycemic control and changes in renal tubular function have been proposed to explain the cardiovascular benefits of SGLT2i. First, the hemodynamic effects of SGLT2i optimize the ventricular loading conditions [150]. SGLT2 inhibition in the proximal tubule results in natriuresis and glycosuria. The ensuing osmotic diuresis reduces the plasma and interstitial fluid volumes which decrease cardiac preload. As a consequence, a reduced afterload may occur via reduced arterial pressure. However, other possible mechanisms, such as improved endothelial function, a decrease in arterial stiffness, a decrease in body weight, and nephron remodeling, may be at work [135,150,151].

Improved blood pressure control is documented in numerous studies. Zelniker et al. observed a reduction of 4–6 mmHg in systolic and 1–2 mmHg in diastolic blood pressure with the use of SGLT2i [151]. Compared with diuretics, SGLT2i cause a greater reduction in interstitial fluid without the risk of volume depletion and hypoperfusion [152].

SGLT2i-associated cardiovascular benefits also depend on improved cardiac metabolism and bioenergy [135,138]. Like T2DM, HF is characterized by a state of insulin resistance [46], where nutrient transport into cardiomyocytes is increased but nutrient utilization is impaired, resulting in energy starvation in the midst of nutrient overabundance [153]. In the insulin-resistant heart, FFAs are favored as a source of energy over glucose [1], even if there is an impairment in mitochondrial nutrient transport, oxidative metabolism, and phosphorylation [153]. These metabolic changes cause insufficient ATP production (reducing cardiac metabolic efficiency), overproduction of reactive oxygen species, and induction of cardiomyocyte steatosis and hypertrophy with the deposition of triacylglycerols. SGLT2i promote ATP production and restore normal cellular metabolism by increasing nutrient-deprivation signaling and normalizing nutrient transport in cardiomyocytes [153]. These drugs may also provide alternative energy sources for the heart by increasing the circulating levels of ketone bodies and/or glucagon secretion [134]. Some studies suggest that SGLT2i may also reverse mitochondrial dysfunction, accelerating the disposal of dysfunctional mitochondria and promoting mitochondrial biogenesis [153]. Overall, metabolic changes mediated by SGLT2i seem to have a positive effect on LV remodeling, mechanical efficiency, and diastolic function [154].

Taken together, SGLT2i reverse the profound nutrient, metabolic, and cellular signaling abnormalities seen in HF, thereby restoring the myocardium to a molecular and cellular phenotype that resembles that of a healthy adult heart.

## 6. Future Perspectives

The cardiac metabolism is a complex system of interconnected pathways that work together to improve the function of the heart. Due to a lack of substrate and post-transcriptional enzyme modification, this ideal system tends to become out of sync in failing hearts, and particularly in DCM. This causes some pathways to be enhanced while others are diminished, which results in less efficient energy production. A negative change impacting the advanced stages of HF is a deficit in mitochondrial capacities, particularly in oxidative capacities.

To decrease or even stop HF metabolic derangement, a number of treatment approaches have been suggested, including increasing ketone body oxidation, increasing glucose oxidation and boosting insulin sensitivity, changing fatty acid oxidation, and increasing BCAA oxidation. Many compounds disappointed expectations when tested on HF patients; they displayed inconsistent findings in terms of heart functions or survival outcomes, despite the pre-clinical investigations starting with promising results. It remains to be determined and researched in additional studies whether these unfavorable results are attributable to a poor sample selection (due, for instance, to the severity of HF) or to many and diverse mechanisms that have not yet been fully clarified. The most successful strategy so far appears to include changing how the failing heart uses its substrates while also utilizing a number of pharmacological medications. The majority of the current research findings are consistent with the idea that an effective supplementary treatment for individuals with HF could involve switching the energy substrate preference away from fatty acid metabolism and toward enhanced myocardial glucose consumption. Nevertheless, further extensive multicenter randomized studies are required before the precise role of metabolic treatment in HF can be determined.

## Figures and Tables

**Table 1:** Positive metabolic effects of cardiovascular drugs in dilated cardiomyopathy. Abbreviations: ARNI, angiotensin receptor neprilysin inhibitor; FFA, free fatty acid; MRA, mineralocorticoid receptor antagonists; RAAS, renin–angiotensin–aldosterone system; SGLT2i, sodium-glucose cotransporter-2 inhibitors; 3-KAT, 3-ketoacyl CoA thiolase.

Betablockers	Reduced circulating FFA levels. Decreased peripheral lipolysis. Increased carbohydrate utilization. Improved insulin sensitivity (carvedilol).
RAAS inhibitors	Enhanced skeletal muscle blood flow, increase in bradykinin, or more effective insulin release to improve glucose homeostasis.
MRAs	Increased cortisol level has detrimental effects on glucose and lipid homeostasis through blockage of glucocorticoid receptors (eplerenone appears to have no metabolic effects).
ARNI	More efficient glucose metabolism.
Trimetazidine	Selective inhibition of the last beta-oxidation enzyme, 3-KAT, activity. Decreased FFA oxidation. Improved insulin sensitivity. Enhanced glucose oxidation and glycolysis.
Ranolazine	Modulation of the late sodium current to prevent the buildup of intracellular Ca++. Enhanced oxidation of glucose.
Etomoxir	Modulation of late sodium current, preventing the buildup of intracellular Ca++ via upregulating a number of beta-oxidation-related enzymes. Enhanced oxidation of glucose.
SGLT2i	Enhanced ketogenesis and glucose absorption in muscle cells. Improved insulin sensitivity. Increased urine glucose excretion. Gluconeogenesis reduction.

## Data Availability

Not applicable.
